# Juvenile Fibroadenoma in a Four-Month-Old Male Infant

**DOI:** 10.5334/jbsr.2155

**Published:** 2020-07-22

**Authors:** Steven Van den Berge, Machteld Keupers, Luc Breysem

**Affiliations:** 1 UZ Leuven, BE

**Keywords:** juvenile fibroadenoma, phyllodes tumor, ultrasound, MRI, pediatric breast mass

## Abstract

**Teaching point:** As juvenile fibroadenoma is radiologically indistinguishable from a (malignant) phyllodes tumor, a core biopsy is decisive for both treatment and follow-up.

## Case report

A four-month-old male infant was referred to our tertiary center for further investigation of a rapidly growing mass in the right breast. On clinical examination, a rounded lesion was palpable, attached to the areola-nipple complex but not to the chest wall, with discoloration of the overlying skin and distention of the superficial veins.

**Figure 1:**
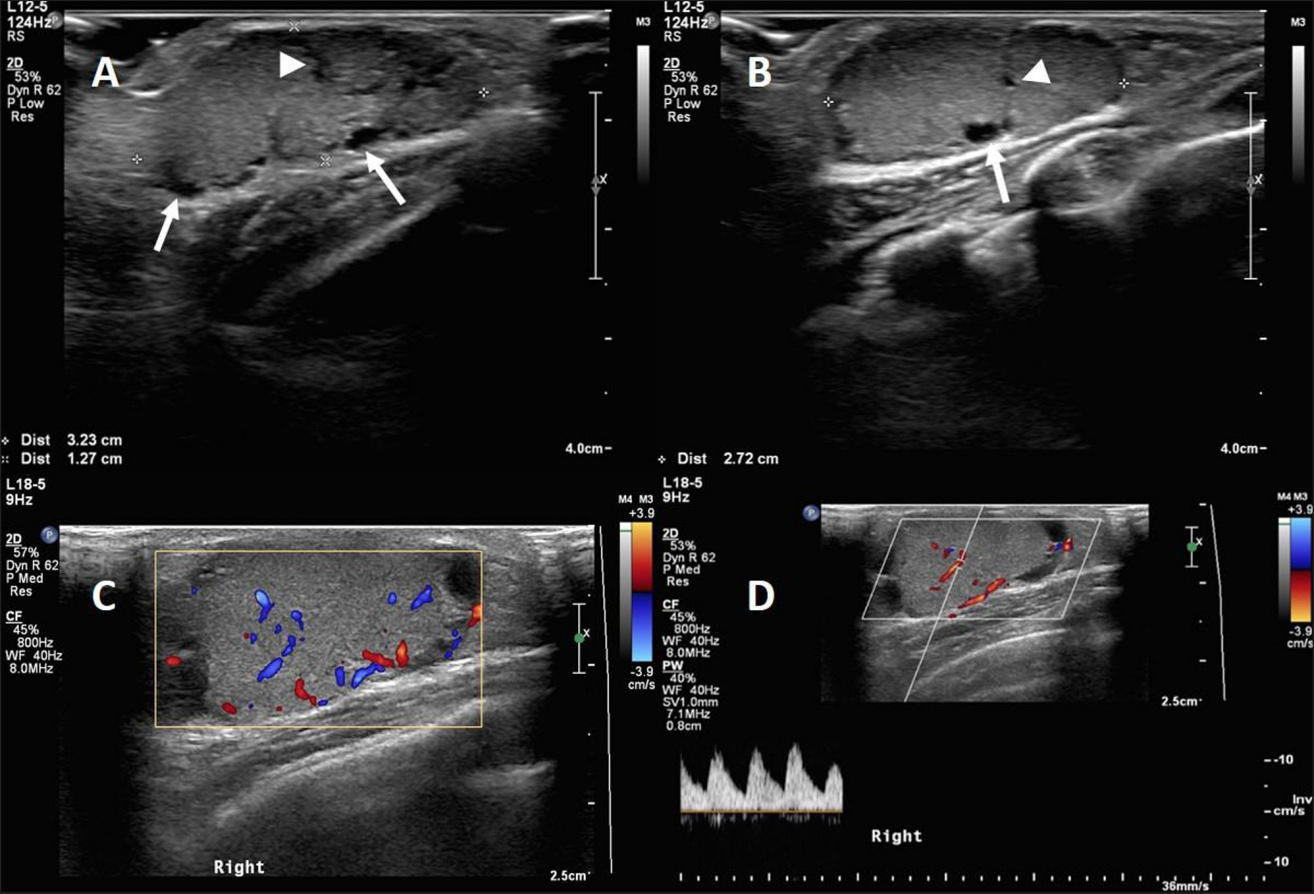
Ultrasound examination **(A–D)** demonstrates a well-defined, heterogenous lesion (3,2 cm LL × 1,3 cm AP × 2,7 cm CC) with sharp boundaries. Note the peripheral cysts (arrows) and hypoechoic clefts (arrowheads) (A–B). The mass is vascularized (C–D), predominantly peripheral, with detectable arterial flow.

Ultrasound (US) (Figure 1A–D) was performed and demonstrated an oval, heterogeneous lesion with sharp boundaries. In the periphery of the lesion, multiple anechoic cysts (arrows) were present. Moreover, it appeared there were some internal hypoechoic clefts (arrowheads) (Figure 1A–B). The mass was highly vascularized with detectable arterial flow (Figure 1C–D).

**Figure 2:**
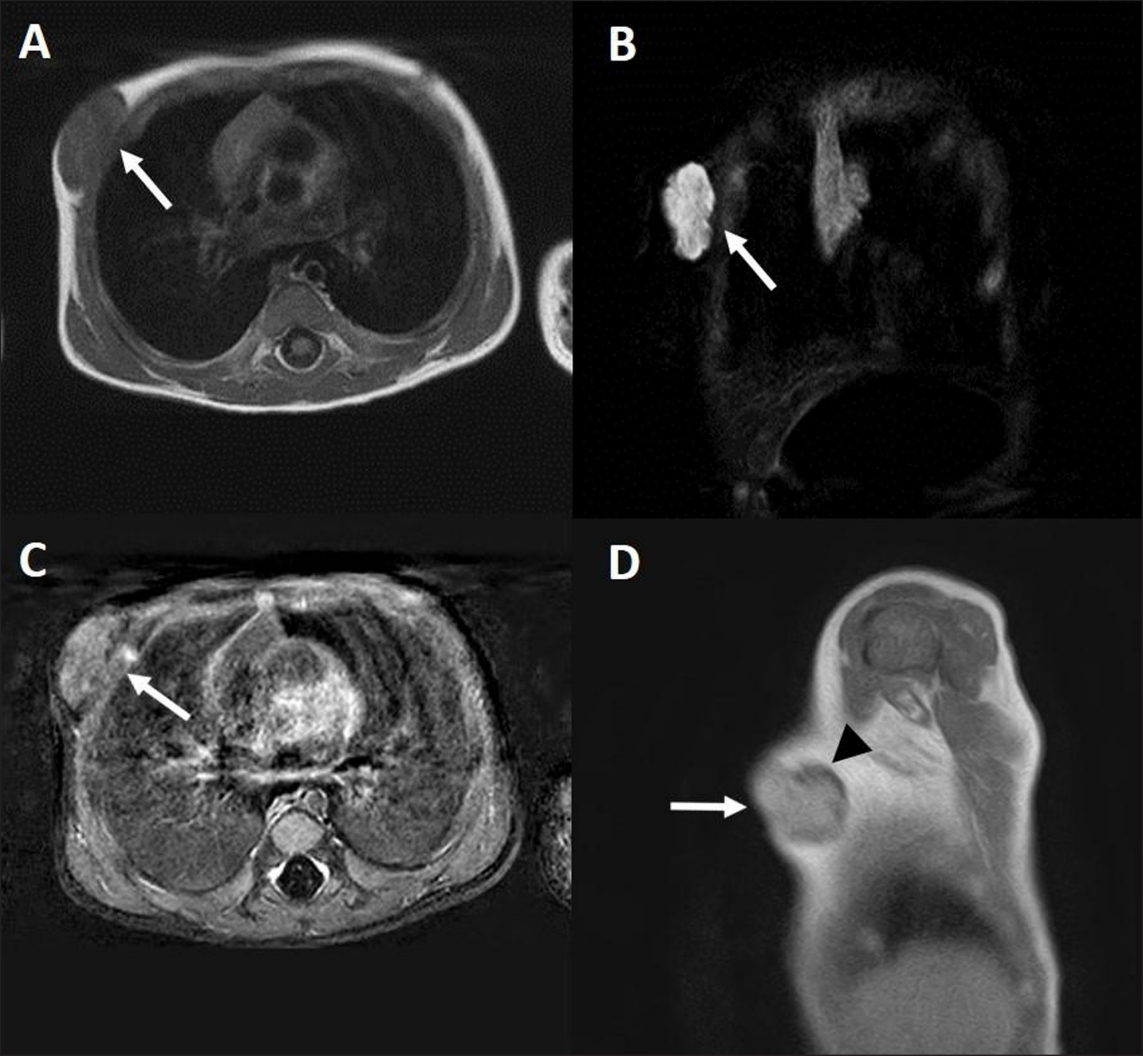
MRI examination (A-D) with axial T1 **(A)**, coronal STIR **(B)**, axial T1-substraction **(C)** and sagittal T1 post-contrast **(D)** images. The mass is highlighted with arrows. Note the non-enhancing peripheral cysts (arrowhead) on image D.

**Figure 3:**
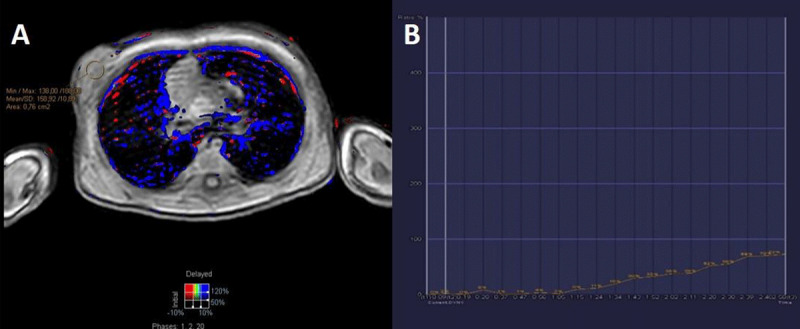
Axial dynamic THRIVE sequence **(A)** illustrating a type 1 kinetic enhancement curve **(B)**.

Additional magnetic resonance (MR) examination (Figure 2A–D) showed a well-defined, T1 isointense (Figure 2A) and Short-Tau Inversion Recovery (STIR) hyperintense (Figure 2B) mass (arrows) superficial to the pectoralis muscle. Axial T1 post-contrast subtraction (Figure 2C) and sagittal T1 post-contrast (Figure 2D) images demonstrated a homogeneous enhancement of the lesion with the exception of the peripherally located cysts (arrowhead). Axial dynamic imaging illustrated a type 1 kinetic curve within the lesion (Figure 3A–B). There was no restricted diffusion.

To obtain diagnostic certainty, a core biopsy was performed, which confirmed the diagnosis of juvenile fibroadenoma.

## Comment

A juvenile or cellular fibroadenoma is an uncommon histologic variant that constitutes approximately 7%–8% of all fibroadenoma subtypes. They most often occur in African American adolescent girls and are characterized by a markedly rapid growth [1]. The incidence of this rare variant is almost nonexistent in male infants. To our knowledge, no such case has been described in the literature to date.

Imaging of these lesions is most commonly accomplished with ultrasound. A hypoechoic solid mass with circumscribed margins, oval morphology and parallel orientation to the chest wall are typical US features of fibroadenoma. The presence of (micro)cysts, hypoechoic clefts or increased vascularity is atypical, though may help differentiate a juvenile from a classic fibroadenoma. These atypical features may also occur in entities as juvenile papillomatosis or phyllodes tumor. It is important to acknowledge this radiological similarity as the latter may be malignant and juvenile papillomatosis portends a poorer prognosis due to its association with malignancy and atypia [1]. MR is of added value for tissue differentiation but is unable to distinguish with certainty a juvenile fibroadenoma from a (malignant) phyllodes tumor. Certain MRI characteristics such as low ADC-values or decreased T2 signal intensity are associated with malignancy, but their sensitivity and specificity are rather low. Therefore, a core biopsy should always be performed to exclude malignancy and to plan for any surgery. A juvenile fibroadenoma can be clinically monitored or treated by simple enucleation, unlike a phyllodes tumor which requires a wide excision due to the increased risk of local recurrence.

Besides (malignant) phyllodes tumor and juvenile papillomatosis, pseudoangiomatous stromal hyperplasia (PASH) should be considered in the radiological differential diagnosis [1]. All of these entities are extremely rare in the pediatric male population and are inconsistent with a clinician’s differential diagnosis including gynecomastia and vascular malformations.
